# Symptomatic pes planus in children: a synthesis of allied health professional practices

**DOI:** 10.1186/s13047-020-0372-8

**Published:** 2020-01-23

**Authors:** Stewart C. Morrison, Madeleine Tait, Elaine Bong, Kyra J. Kane, Chris Nester

**Affiliations:** 1 University of Brighton, Eastbourne, UK; 2 Wascana Rehabilitation Centre, Saskatchewan Health Authority, Saskatchewan, Canada; 3 School of Health and Society, University of Salford, Salford, UK

**Keywords:** Paediatric, Orthoses, Orthotist, Physiotherapist, Podiatrist, Feet

## Abstract

**Background:**

This study sought to explore professional perspectives on the assessment and management of symptomatic pes planus in children.

**Methods:**

Data was collected from three professional groups (podiatrists, physiotherapists, and orthotists) with experience of managing foot problems in children. The survey was undertaken in the United Kingdom via a self-administered, online survey. Data was captured over a four-month period in 2018.

**Results:**

Fifty-five health professionals completed the survey and the results highlighted that assessment techniques varied between professions, with standing tip-toe and joint range of motion being the most common. Treatment options for children were diverse and professionals were adopting different strategies as their first line intervention. All professions used orthoses.

**Conclusions:**

There were inconsistencies in how the health professionals assessed children presenting with foot symptoms, variation in how the condition was managed and differences in outcome measurement. These findings might be explained by the lack of robust evidence and suggests that more effort is needed to harmonise assessment and treatment approaches between professions. Addressing discrepancies in practice could help prioritise professional roles in this area, and better support the management of children with foot pain.

## Background

Paediatric pes planus (flat foot) is a nebulous presentation which has challenged clinicians for many years [1–5]. Pes planus is typically described as flexible (non-osseous) or fixed (osseous) [6] and is a common presentation to clinical services [7–9]. The flexible variant can be further characterised as asymptomatic (often referred to as physiological) or symptomatic [6]. Typical features of symptomatic pes planus include foot pain [1, 6, 10], functional impairment such as tripping and fatigue [1], proximal joint problems [11], and reduced quality of life [12, 13].Children with both asymptomatic and symptomatic pes planus frequently present to health professionals with parents reporting concerns about the physical appearance of their child’s feet [7, 14]; however opinion supports intervention for symptomatic presentations only [1, 4, 6].

Pes planus is often characterised by observable features of foot shape and position (e.g., decreased height of the medial longitudinal arch, and valgus position of the rearfoot), although there are no standardised criteria [2, 15, 16]. Given this ambiguity it is no surprise that evidence underpinning treatment effectiveness remains elusive [17]. Treatment for the symptomatic presentation is often multi-faceted and includes non-surgical [17, 18] and surgical [3, 6, 8] interventions. Common non-surgical interventions include footwear advice, stretching and strengthening exercises, and foot orthoses [6], yet there is little guidance on what an appropriate intervention is, and when, or for whom, this should be used. Research amongst Canadian physiotherapists identified a lack of consensus regarding clinical use of foot orthoses for children with gross motor delay [10]. More recent work by Dars and colleagues [1] sought consensus on diagnostic methods and interventions for children with flexible pes planus; however this only considered the perspective(s) of podiatrists. Within the UK, foot orthoses are commonly prescribed by podiatrists, physiotherapists, and orthotists [19] and therefore, further work is needed to understand how these professions approach assessment and management of children with pes planus.

The aim of this work was to explore health professional (podiatrist, physiotherapist and orthotist) perspectives on the assessment and management of symptomatic pes planus in children. Given the persistent debate about this condition, exploring current practices may help ensure consistent, evidence-based assessment and intervention strategies are delivered by health care professionals working in this area.

## Methods

### Design

This was a descriptive study which sought to understand the common approaches that health professionals adopt when considering the assessment and management of children with symptomatic pes planus.

### Survey development

A cross-sectional, self-administered UK based online survey was developed to enable access to a broad reach of allied health professionals with clinical experience of managing foot problems in children. This was an open survey and data collection was undertaken across a four-month period from July – October 2018. Prior to data collection, a favourable opinion was provided by the ethics committee within the School of Health Sciences at the University of Brighton.

An initial draft of the survey was aligned with an existing survey tool [10]. Consultation with clinicians and academics with experience in paediatric physiotherapy and podiatry was undertaken and additional questions developed. The first draft was piloted on three academics within the physiotherapy and podiatry professions. This focussed on the relevance of the questions, accuracy of the terminology and overall structure. The final survey comprised 18 questions across four sections and both adaptive and mandatory questions were used in the survey. There were eight open-ended questions, five closed questions (multiple-choice or dichotomous) and five which combined both (See Additional file 1). Open-ended and fixed questions that had a choice of “other” allowed participants a free-text answer.

The first section collected demographic information, including profession, and percentage caseload (paediatrics). The second section focussed on clinical characteristics and assessment of pes planus. The third section focussed on intervention(s) and sought to elicit whether orthoses were used as first-line interventions, common indications for prescribing foot orthoses, parent expectations, and the types(s) of orthoses commonly used. The final section considered the broader aspects of clinical management such as side-effects from use of orthoses, outcome measures and timeline(s) for intervention.

The survey was administered using Online Surveys (onlinesurveys.ac.uk). All participants read an overview of the project via the online information sheet and provided informed consent via a statement embedded within the survey.

### Participant recruitment

Podiatrists, physiotherapists, and orthotists registered with the Health and Care Professions Council (HCPC) and with experience of treating symptomatic pes planus in children under 16 years old, working within the UK, were invited to participate. Specialist paediatric advisory groups representing the professions were contacted by email and asked to disseminate the survey via their social media platforms. Invitations for participation were also shared via social media platforms (i.e. Twitter) with hyperlinks to background information about the study, consent statement, and survey.

### Data analysis

All survey responses were collated within the online survey platform. Data was then exported to Microsoft Excel (Microsoft Office 2016) and cleaned prior to analysis to ensure respondents satisfied the inclusion criteria (e.g. participants’ county of residence within the UK). Data were analysed using descriptive statistics and grouped by profession. Two of the authors (MT, SM) reviewed the free-text responses and agreed that they did not offer the conceptual richness to be considered for qualitative analysis [20]. Most of the open text responses were limited to a few words and as such, a summative content analysis was applied to the data [21, 22]. An inductive approach to the coding framework was adopted [22]. The responses were organised and then categorised into quantitative units (e.g. converting open text about side-effects from intervention into measurable units) to enable descriptive analysis. The coding, categorisation and synthesis of the data was undertaken by one author (MT).

Odds ratios were calculated to quantify effect size by measuring the association between professions. A ratio of one suggested an equivalent likelihood of the response between the two professions, and more than one meant a greater likelihood of the response in one profession compared to the other. Statistical Package for Social Sciences V25.0 (IBM Corp., New York, USA) was used for statistical analysis.

## Results

### Section 1 - demographics

As data collection was undertaken via an online survey, there was no single response rate [23]. The survey had a view response of 389, a participation rate of 149 and completion rate of 55 responses. Complete responses were received from 33 physiotherapists (60.0%); 16 podiatrists (29.0%); and 6 orthotists (11.0%; Table 1). Eighty-seven percent of these were based in England, 9.3% in Scotland and 3.7% in Wales. Most participants (69%) had been working more than 10 years in their paediatric role, whilst the orthotists were newer to their practice, with 83.3% working less than 5 years.

**Table 1:** Demographics of participants completing the survey

	PT	Pod	Orth
Percentage of respondents from profession(s) (%)	60	29	11
Typical practice (%)			
NHS	93.9	93.5	66.6
PP	–	6.2	16.7
Other	6.1	–	16.7
Paediatric component of caseload (%)			
0–24	30.3	37.5	50
25–49	54.5	31.3	16.7
50–74	9.1	6.3	33.3
75–99	6.1	8.8	–
100	–	6.3	–
Years of experience in profession (%)			
0–5	9.1	6.3	83.3
6–10	18.2	12.5	–
11–20	39.4	50.0	–
≥21	33.3	31.3	16.7
Years of paediatrics experience (%)			
0–5	18.2	12.5	83.3
6–10	21.2	43.8	–
11–20	39.4	37.5	–
≥ 21	21.2	6.3	16.7

*PT* Physiotherapist, *Pod* Podiatrist, *Orth* Orthotist, *NHS* National Health Service, *PP* private /Independent practice

Fifteen (94%) of the podiatrists worked in the NHS and of these, 14 (87%) were based in England. Thirty-one (94%) of the physiotherapists worked in the NHS and of these, 24 were based in England, 4 in Scotland and 2 in Wales. Four of the orthotists worked in the NHS and one in private practice. All were based in England.

### Section 2 - clinical characteristics and assessment

Across the three professions, standing tip-toe and joint range of motion were the most frequent assessment techniques (Table 2) used. Podiatrists most often assessed joint range of motion (68.8%), foot shape (FPI-6) (62.5%) and standing tip-toe (62.5%), whilst physiotherapists preferred standing tip-toe (51.5%) over joint range of motion (36.4%). Orthotists most frequently assessed joint range of motion (83.3%), muscle power (83.3%), and standing tip-toe (66.7%). Compared to physiotherapists, podiatrists were 6.9 times more likely to use the FPI-6 (62.5% versus 9.1%) and 5.1 times more likely to assess single leg balance (31.3% versus 6.1%). There was no apparent consensus on the factors that predisposed children to developing symptoms. The responses were multi-factorial and included: neurodevelopmental and motor control issues, muscle weakness, limited joint range of motion, tissue stress, obesity, hypermobility, poor footwear and parent concern/anxiety.

**Table 2:** Clinical Assessment of Pes Planus

Assessment	Profession (%)			Odds ratio		
	Pod	PT	Orth	Pod versus Orth	Pod versus PT	PT versus Orth
FPI-6	62.5	9.1	33.3	1.9	6.9	0.3
Standing tip-toe	62.5	51.5	66.7	0.9	1.2	0.8
Neurological tests	50.0	27.3	–		1.8	
Joint range of movement	68.8	36.4	83.3	0.8	1.9	0.4
History taking	31.3	12.1	16.7	1.9	2.6	0.7
Hubshur/Jacks test	37.5	33.3	–		1.1	
Patient complaint	12.5	18.2	16.7	0.7	0.7	1.1
Gait analysis	18.8	21.2	16.7	1.1	0.9	1.3
X-Ray imaging	12.5	9.1	16.7	0.7	1.4	0.5
Single leg balance	31.3	6.1	50.0	0.6	5.1	0.1
Muscle power	18.8	6.1	83.3	0.2	3.1	0.1
Beighton test	12.5	3.0	–		4.2	

FPI-6 Foot Posture Index; *PT* Physiotherapist, *Pod* Podiatrist, *Orth* Orthotist

Joint stiffness or atypical structural features were the most common reasons for x-ray referral (60%). Other considerations included lack of response to conservative treatment (31%), or clinical findings that were indicative of underlying pathology (13%). Podiatrists and physiotherapists were most likely to request x-rays if pain had not resolved after conservative treatment (93.8 and 45.5%), whilst orthotists were likely to request x-ray when severe deformity presented - such as skew foot or greater joint stiffness.

### Section 3 - intervention

Forty percent of all participants reported that foot orthoses were their first-line intervention for symptomatic children (Table 3). Where orthoses were not the first-line intervention, participants were asked to detail their intervention(s). Most of the podiatrists adopted strengthening exercises (62.5%) and footwear advice (50%) as their first line intervention. This was less clear for the physiotherapists where there was a divide between those using orthoses as a first-line intervention (54.5%) and those who were not (45.4%). Use of patient education was the largest difference between professions, with podiatrists using education 4.2 times more often than physiotherapists (12.5% versus 3.0%), and no orthotists reporting use of education.

**Table 3:** Clinical interventions used in the management of pes planus

Intervention	Profession (%)			Odds ratio		
	Pod	PT	Orth	Pod versus Orth	Pod versus PT	PT versus Orth
Orthoses not first line	81.3	54.5	33.3	2.4	1.5	1.6
Orthoses first line	18.8	45.5	66.7	0.3	0.4	0.7
Education	12.5	3.0	–		4.2	
Strengthening exercises	62.5	39.4	33.3	1.9	1.6	1.2
Stretching exercises	18.8	18.2	16.7	1.1	1.0	1.1
Activity modification	18.8	12.1	–		1.6	
Footwear advice	50.0	15.2	16.7	3.0	3.3	0.9
Taping	18.8	–	–			
Nutrition advice	6.3	3.0	–		2.1	
Manipulation	–	3.0	–		–	
Referral for foot orthoses	–	6.1	–		–	

*PT* Physiotherapist, *Pod* Podiatrist, *Orth* Orthotist

Podiatrists tended to use prefabricated orthoses (81%) over bespoke orthotic devices (6%). The physiotherapists used bespoke (22%) and prefabricated devices (18%); the orthotists used prefabricated devices (33%). The choice of prefabricated or bespoke orthoses was dependent on the clinical presentation of the child for 13% of the podiatrists, most of the physiotherapists (60%) and orthotists (67%). Referral from physiotherapy to podiatry for prescription of orthoses was common (39.3%). The factors that clinicians considered when deciding on their choice of orthoses were mixed, and often clinician specific. These included the presentation of the foot and level of ‘control’ required from the orthoses, symptom severity, child’s age, bodyweight, footwear preference, and activity. The age at which clinicians reported prescribing foot orthoses was typically between the ages of 2–5 years, and most commonly aged 3 years and above (as reported by 54% of respondents). Twenty percent of all respondents would consider intervention in children under 2 years of age, whereas 14% would not intervene until the child was older. Seven percent reported no lower age limit and 5% did not respond to the question.

Participants commented on whether they would prescribe orthoses for an asymptomatic child. Fifty-two percent of all clinicians would not treat with orthoses whilst 46% reported that it depended on the child. One podiatrist reported that they would intervene with orthoses. Participants who answered “*depends*” reported several clinical flags which informed their decision making, including severity of foot presentation (e.g. ‘excessive pronation’) (44%), proximal issues (e.g. lower back pain) (20%), gait or functional changes (16%), and hypermobility (12%). Two of the physiotherapists (8%) reported that it was not their decision.

All professional groups indicated that parents expected them to prescribe/provide foot orthoses for their child (Podiatrists - 68.8%; Physiotherapists - 48.5%; Orthotists 83.3%). When seeing an orthotist, footwear as an intervention was 5.3 times more likely to be expected by parents compared to a podiatrist (33.3% versus 6.3%) and 2.2 times more likely compared to physiotherapists (33.3% versus 15.2%).

### Section 4 patient outcomes and treatment side-effects

The most frequently used outcome measures for podiatrists were a VAS (62.5%), patient reported pain (31.3%), and general health questionnaires (31.3%) (Table 4). Physiotherapists most often used patient reported pain (57.6%), gait analysis (30.3%), function (27.3%), and VAS (27.3%). Orthotists reported using patient reported pain (50.0%), VAS (33.3%), general health questionnaires (33.3%), and paediatric-specific questionnaires (33.3%). Physiotherapists and orthotists incorporated more paediatric-specific questionnaires (6.1 and 33.3%), whereas podiatrists tended to incorporate more foot-specific questionnaires (18.8%). Physiotherapists (30.3%) used gait analysis more than podiatrists (6.3%) and orthotists (0.0%). Activity level was used as an outcome measurement by podiatrists (25%) and physiotherapists (6.1%), but not orthotists (0.0%).

**Table 4:** Outcome measures used in practice

Outcome measurement	Profession (%)			Odds ratio		
	Pod	PT	Orth	Pod versus Orth	Pod versus PT	PT versus Orth
Paediatric specific questionnaire	–	6.1	33.3			0.2
Foot specific questionnaire	18.8	–	–			
General health questionnaire	31.3	9.1	33.3	0.9	3.4	0.3
Wong-Baker FACE pain score	12.5	–	–			
Visual analogue score	62.5	27.3	33.3	1.9	2.3	0.8
Patient reported pain	31.3	57.6	50.0	0.6	0.5	1.2
Activity levels	25.0	6.1	–		4.1	
Hubshur/Jacks test	6.3	–	–			
Balance	–	6.3	16.7			0.4
Parental reported complaint	12.5	3.0	16.7	0.7	4.1	0.2
Gait analysis	6.3	30.3	–		0.2	
Functionality	–	27.3	–			
Strengthening exercises	–	6.1	–			
Stamina	–	6.1	–			

*PT* Physiotherapist, *Pod* Podiatrist, *Orth* Orthotist

Participants were asked to select one factor which they considered most important when determining the time to discontinue orthoses. Sixty-three percent reported that resolution of symptoms was the key marker for withdrawal of orthoses, but the free text comments acknowledged that the decision-making process was complex. Some participants considered motor skills (7%), foot shape (4%), family factors (4%), and other factors (22%).

The most frequent side-effects from foot orthoses included localised skin irritation (e.g. blisters) (25%) and increased pain (25%). Problems with shoe-fit (22%) and intolerance/discomfort (11%) were also highlighted. Some respondents did not report that their patients experienced side-effects (23%).

## Discussion

The findings from this survey offer a snapshot of professional approaches to assessment and management of symptomatic pes planus, and highlight common strategies adopted by the three professional groups. The results suggest that these professional groups have differing priorities for clinical assessment and demonstrate some inconsistencies with the approaches to intervention and use of outcome measures. These results might help to contribute to wider efforts seeking to better understand the working context and scope of practice of health professionals who prescribe foot orthoses [19, 24].

Assessment techniques for symptomatic pes planus varied between professions and diverged from recent recommendations advocating use of the FPI-6 and static, footprint-based measures [16]. None of the respondents reported using static footprint measures, but many included both static and dynamic assessments; this was most commonly joint range of motion and standing tip-toe, although the podiatrists also used the FPI-6. Assessment of foot shape was a priority for the podiatrists but all professions appeared to make treatment decisions based on physical presentation and (to some extent) physical function, as supported by the existing literature [1]. Further consideration of the theoretical frameworks that inform assessment choices may clarify the relationship between these approaches and the interventions that are subsequently selected. Based upon the findings from this survey, it is unclear if the assessment techniques resulted in different outcomes.

The data demonstrated that treatment options were diverse, and professionals adopted various first-line approaches. Orthoses, stretching and/or strengthening exercises, footwear advice, and complementary strategies (e.g. manipulation) were all identified as components of care and these mixed practices are broadly consistent with the existing literature [8, 17]. Foot orthoses were commonly used by all three professions, despite debate over their effectiveness [17] and robust evidence remaining elusive. As expected, the data suggested that the professional groups used orthoses in different ways (accompanied or augmented by a variety of other intervention modalities). The results also highlight some early evidence that parents have different expectations of the professional groups, which suggests that it is important to understand more about the profile of the children who are seeing the different professionals. Consistent with previous reports [1], the podiatrists in this study preferred prefabricated orthoses to bespoke devices; but there was variability across the professions. Disagreement about orthoses as a first-line intervention was most evident amongst the physiotherapists. These descriptions of intra- and inter-professional practices echo a wider discussion about the quality and consistency of orthotic service provision within musculoskeletal services [25]. It is clear that clinical approaches are multi-factorial and likely to represent a combination of geographical factors [26], sparse evidence [17], and professional preferences [27] . It is feasible that lack of knowledge and/or confidence may also impact consistency in practice [28], and further evidence to inform management strategies is needed.

The survey also sought to understand how clinicians responded to the asymptomatic children referred into their services. Consistent with previous results [7], most clinicians in this sample indicated that they would not intervene. The data suggested that various factors, most commonly foot presentation, informed clinical decision-making and this implies that some clinicians would intervene if they felt the foot presentation was “*excessive*” or in some way outside typical boundaries. Efforts are needed to ensure that paediatric interventions are underpinned by appropriate conceptual frameworks such as the International Classification of Functioning, Disability and Health (ICF) [29]. Respondents to this survey reported that they used few objective, standardised measures to evaluate outcomes in practice. Whilst the use of VAS and (non-specific) patient reported outcomes were common, a shift towards disease-specific assessment and consideration of functional capabilities is indicated. Efforts to adopt developmentally-appropriate [30], validated, child-centred outcome measures (such as the Oxford Ankle Foot Questionnaire) [31], which are aligned to patient goals, might complement existing measures. Ensuring that outcome measures are aligned with the ICF [29] domains, particularly the activity and participation dimensions, by emphasizing activity and participation-related outcomes rather than impairments of body structure and function could help foster a more biopsychosocial approach to intervention and facilitate integrated services for these children. This could also help erode some of the persisting (but out-dated) approaches to assessment and intervention for these children.

Whilst the results of this survey help to inform our understanding of current approaches to the assessment and management of symptomatic (flexible) pes planus, the findings must be considered in light of some limitations. We adopted an open invitation sampling approach of clinicians with the UK and this was facilitated via social media and professional networks. Inevitably, this introduced a volunteer bias and the findings may not fully represent the professions recruited into this study. Further, the sample size was moderate overall and small for orthotists, which reduces the external validity of the findings and impacts on comparison between professional groups. To the best of our knowledge, there is no publicly available workforce data which details the percentage of the professions working in paediatrics within the UK. Therefore, we are unable to define the target population or explore the extent to which the survey respondents represent paediatric specialists within each of the professions. We also acknowledge that the external validity of the work is compromised as our sampling was limited to the UK.

## Conclusion

This survey described the approaches of a group of UK-based podiatrists, physiotherapists, and orthotists to the clinical assessment and management of symptomatic, flexible pes planus in children. The findings demonstrated variation in assessment and intervention approaches for this population, both between and within professions. Foot orthoses were a common first-line strategy adopted by all groups, although there was variability within each profession. Further work is needed to better understand clinical decision-making and evidence-informed approaches to assessment and treatment of pes planus. More consistent, evidence-based strategies underpinned by a child-centred biopsychosocial approach to intervention may help improve outcomes for this group of children.

## Supplementary information

**Additional file 1.** Survey Used for Data Collection.

## Data Availability

The datasets used and/or analysed during the current study are available from the corresponding author on reasonable request.

## Abbreviations

FPI-6: Foot Posture Index – 6
HCPC: Health and Care Professions Council
ICF: International Classification of Functioning, Disability and Health
NHS: National Health Service
Orth: Orthotist
Pod: Podiatrist
PT: Physiotherapist
UK: United Kingdom

## References

[1] Dars S, Uden H, Kumar S, Banwell HA (2018). When, why and how foot orthoses (FOs) should be prescribed for children with flexible pes planus: a Delphi survey of podiatrists. PeerJ..

[2] Carr JB, Yang S, Lather LA (2016). Pediatric Pes Planus: a state-of-the-art review. Pediatrics..

[3] Dare DM, Dodwell ER (2014). Pediatric flatfoot: cause, epidemiology, assessment, and treatment. Curr Opin Pediatr.

[4] Evans AM (2008). The flat-footed child -- to treat or not to treat: what is the clinician to do?. J Am Podiatr Med Assoc.

[5] Cappello T, Song KM (1998). Determining treatment of flatfeet in children. Curr Opin Pediatr.

[6] Harris EJ, Vanore JV, Thomas JL, Kravitz SR, Mendelson SA, Mendicino RW (2004). Diagnosis and treatment of pediatric flatfoot. J Foot Ankle Surg.

[7] Yeo A, James K, Ramachandran M (2015). Normal lower limb variants in children. BMJ..

[8] Halabchi F, Mazaheri R, Mirshahi M, Abbasian L (2013). Pediatric flexible flatfoot; clinical aspects and algorithmic approach. Iran J Pediatr.

[9] Pfeiffer M, Kotz R, Ledl T, Hauser G, Sluga M (2006). Prevalence of flat foot in preschool-aged children. Pediatrics..

[10] Kane K (2015). Foot orthoses for pediatric flexible flatfoot: evidence and current practices among Canadian physical therapists. Pediatr Phys Ther.

[11] Kothari A, Dixon PC, Stebbins J, Zavatsky AB, Theologis T (2016). Are flexible flat feet associated with proximal joint problems in children?. Gait Posture.

[12] Kothari A, Stebbins J, Zavatsky AB, Theologis T (2014). Health-related quality of life in children with flexible flatfeet: a cross-sectional study. J Child Orthop.

[13] López López D, Bouza Prego ML, Requeijo Constenla A, Saleta Canosa JL, Bautista Casasnovas A, Tajes FA (2014). The impact of foot arch height on quality of life in 6-12 year olds. Colomb Med (Cali).

[14] Carli A, Saran N, Kruijt J, Alam N, Hamdy R (2012). Physiological referrals for paediatric musculoskeletal complaints: a costly problem that needs to be addressed. Paediatr Child Health.

[15] Sheikh Taha AM, Feldman DS (2015). Painful Flexible Flatfoot. Foot Ankle Clin.

[16] Banwell HA, Paris ME, Mackintosh S, Williams CM (2018). Paediatric flexible flat foot: how are we measuring it and are we getting it right? A systematic review. J Foot Ankle Res.

[17] Dars S, Uden H, Banwell HA, Kumar S (2018). The effectiveness of non-surgical intervention (foot Orthoses) for paediatric flexible pes planus: a systematic review: update. PLoS One.

[18] Evans AM, Rome K (2011). A Cochrane review of the evidence for non-surgical interventions for flexible pediatric flat feet. Eur J Phys Rehabil Med.

[19] Nester CJ, Graham A, Martinez-Santos A, Williams AE, McAdam J, Newton V (2017). National profile of foot orthotic provision in the United Kingdom, part 1: practitioners and scope of practice. J Foot Ankle Res.

[20] O'Cathain A, Thomas KJ (2004). “Any other comments?” open questions on questionnaires - a bane or a bonus to research?. BMC Med Res Methodol.

[21] Hsieh HF, Shannon SE (2005). Three approaches to qualitative content analysis. Qual Health Res.

[22] Kondracki NL, Wellman NS, Amundson DR (2002). Content analysis: review of methods and their applications in nutrition education. J Nutr Educ Behav.

[23] Eysenbach G (2004). Improving the quality of web surveys: the checklist for reporting results of internet E-surveys (CHERRIES). J Med Internet Res.

[24] Nester CJ, Graham A, Martinez-Santos A, Williams AE, McAdam J, Newton V (2018). National profile of foot orthotic provision in the United Kingdom, part 2: podiatrist, orthotist and physiotherapy practices. J Foot Ankle Res..

[25] NHS England (2015). Improving the Quality of Orthotics Services in England.

[26] Morris C, Newdick H, Johnson A (2002). Variations in the orthotic management of cerebral palsy. Child Care Health Dev.

[27] Kane K, Manns P, Lanovaz J, Musselman K. Clinician perspectives and experiences in the prescription of ankle-foot orthoses for children with cerebral palsy. Physiother Theory Pract. 2019;35(2):148–56. 10.1080/09593985.2018.1441346 29465276

[28] Kane KJ, Lanovaz JL, Musselman KE. Physical Therapists’ use of evaluation measures to inform the prescription of ankle-foot Orthoses for children with cerebral palsy. Phys Occup Ther Pediatr. 2019;39(3):237–53. 10.1080/01942638.2018.1463586 29702012

[29] World Health Organisation (2001). International Classification of Functioning, Disability and Health (ICF): Children and Youth Version.

[30] Morrison SC, McClymont J, Price C, Nester C (2017). Time to revise our dialogue: how flat is the paediatric flatfoot?. J Foot Ankle Res..

[31] Morris C, Liabo K, Wright P, Fitzpatrick R (2007). Development of the Oxford ankle foot questionnaire: finding out how children are affected by foot and ankle problems. Child Care Health Dev.

