# Supplementary material for: Symptomatic pes planus in children: a synthesis of allied health professional practices
Source: J Foot Ankle Res. 2020 Jan 23;13:5. doi: 10.1186/s13047-020-0372-8 (PMC6979376; doi:10.1186/s13047-020-0372-8)
Supplement: Supplementary file 1 — Additional file 1. Survey Used for Data Collection. [file 13047_2020_372_MOESM1_ESM.pdf]

Page 2: Demographics

Please confirm your profession by ticking one of the boxes below: ✱ *Required*

- Podiatrist
- Physiotherapist
- Orthotist

What is your main place of clinical work (>50% of your time) ? ✱ *Required*

- NHS
- Private Practice
- Other

If you selected Other, please specify:

How much of your caseload is related to assessment and managment of foot problems in children? ✱ *Required*

- 100%
- 75 - 99%
- 50 - 74%
- 25 - 49%
- 0- 24 %

Where are you based?

- England
- Scotland
- Northern Ireland
- Wales

In total, how many years have you been practicing within your profession? ✱ *Required*

- 0-5 years
- 6 - 10 years
- 11 - 20 years
- 21 + years

How many years have you had a paediatric caseload / role?

- 0-5 years
- 6 - 10 years
- 11 - 20 years
- 21 + years

Page 3: Clinical characteristics & assessment of symptomatic pes planus

Do you agree with the ACFAS definiton of symptomatic pes planus? Here is a link to the guideline https://www.acfas.org/uploadedFiles/Healthcare_Community/Education_and_Publications/Clinical_Practice_Guidelines/ACFAS_Pediatric_Flatfoot_CPG_1.

- Yes
- No
- Partly

If no, why not? Please explain.

What are the assessments that you use to determine if a child has flexible [symptomatic] pes planus? ✱ *Required*

What factors do you think contribute to the onset of symptoms in this patient group? ✱ *Required*

When would it be appropriate to request imaging (e.g. x-ray) as part of your assessment?

If you see a child with non-symptomatic [flexible] pes planus, would you treat them with foot orthoses? ✱ *Required*

- Yes
- No
- Depends

What factors influence your decision-making?

If yes - what are your indications for treatment? Do any guidelines inform your decision-making?

What are the expectations of the parental caregivers when they come to see you in clinic? Do they expect any particular intervention?

Page 4: Orthotic intervention for symptomatic pes planus

Foot orthoses (bespoke or pre-fabricated) are a popular non-surgical intervention for paediatric flexible pes planus, although their effectiveness still remains unclear. Evidence supporting the beneficial impact of orthotics has increased for the management of pain, foot posture, gait and structural function for paediatric pes planus (Dars, Uden, Banwell & Kumar, 2018).

Are foot orthoses your first-line intervention for symptomatic [flexible] pes planus? ✱ *Required*

- Yes
- No

If not, what is your first line intervention?

What type(s) of orthoses do you most commonly use?

- Bespoke, casted
- Prefabricated
- Depends

Please explain your answer

Please describe any modifications that you would commonly make to your foot orthoses.

What is the youngest age you would consider orthotic intervention in a child with symptomatic [flexible] pes planus?

What outcome measures do you use to demonstrate the effectiveness of your intervention? ✱ *Required*

Have your patients reported any side-effects from wearing foot orthoses?

How do you determine when a child no longer needs the foot orthoses and can discontinue wearing them?

- Gross motor skills reach age-appropriate level
- Foot shape reaches normal appearance for age
- Child stops attending therapy
- Symptoms resolve
- Family decision (too much effort, cost, etc.)
- Other

Please explain.

What percentage of any improvement in the child's symptoms is attributable to the orthotic device?

What other strategies form part of your management (e.g. exercise prescription)?

Is there anything else regarding symptomatic [flexible] pes planus that you would like to mention?

Page 5: Survey Finished

We appreciate your time for completing this survey. Thank you very much.
